# Supplementary material for: Investigating the bacterial community of gray mangroves (Avicennia marina) in coastal areas of Tabuk region
Source: PeerJ. 2024 Oct 18;12:e18282. doi: 10.7717/peerj.18282 (PMC11493069; doi:10.7717/peerj.18282)
Supplement: Supplemental Information 1 — Figure S1 photo credit: Hanaa Ghabban. Figure S2 photo credit: Doha A. Albalawi. [file peerj-12-18282-s001.docx]

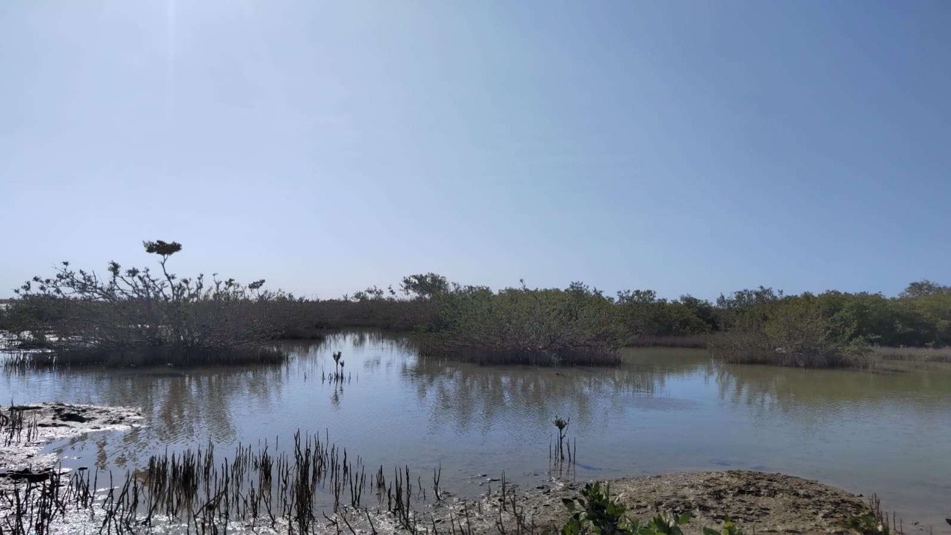


Ras Alshabaan-Umluj (Umluj(, Tabuk region, Saudi Arabia (photos taken by co-author)


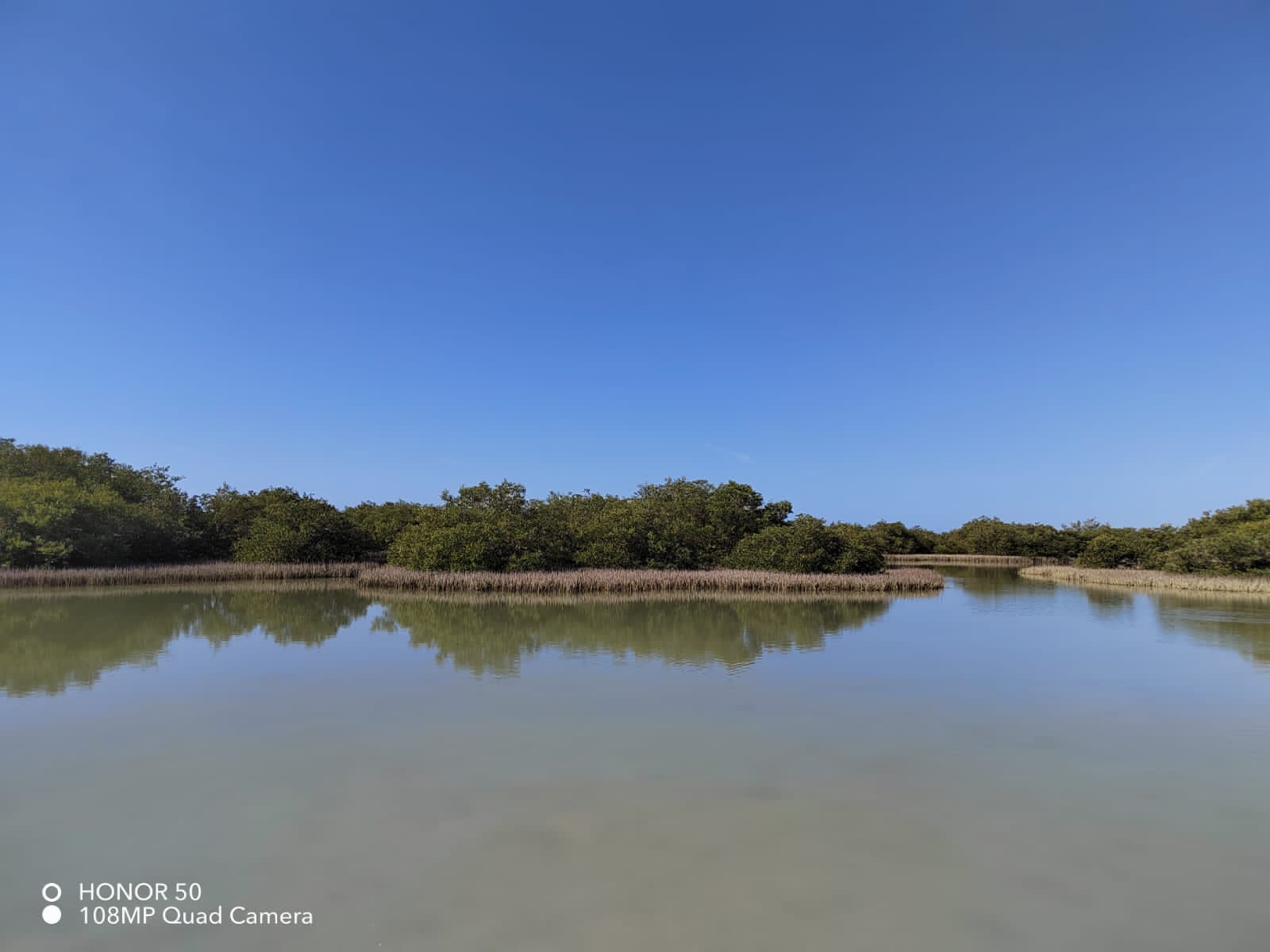


Almunibrah-Al-Wajh (Al-Wajh), Tabuk region, Saudi Arabia (photos taken by co-author)

**Supplementary information Figure S1:**

The sampling sites Ras Alshabaan-Umluj (Umluj( and Almunibrah-Al-Wajh (Al-Wajh) covered under gray mangrove (Avicennia marina) vegetation. The exact coordinates of sampling locations are:

- The bulk soil from Umluj (24°48'11.3"N 37°10'50.6"E), (24°48'10.9"N 37°10'51.2"E), and (24°48'10.5"N 37°10'51.3"E)
- The rhizosphere soil from Umluj (24°48'20.6"N 37°10'45.1"E), (24°48'10.5"N 37°10'49.6"E) and (24°48'10.4"N 37°10'50.0"E)
- The bulk soil from Al-Wajh (26°01'38.4"N 36°42'26.1"E) and (26°01'38.2"N 36°42'26.6"E)
- The rhizosphere soil from Al-Wajh (26°01'36.8"N 36°42'24.4"E(, (26°01'35.4"N 36°42'25.4"E), and (26°01'35.2"N 36°42'25.6"E)

bulk soil and rhizosphere samples were randomly collected from two different areas Umluj (24º47’59” N 37º10’47” E) and Al-Wajh (26º01’30” N 36º42’31” E)


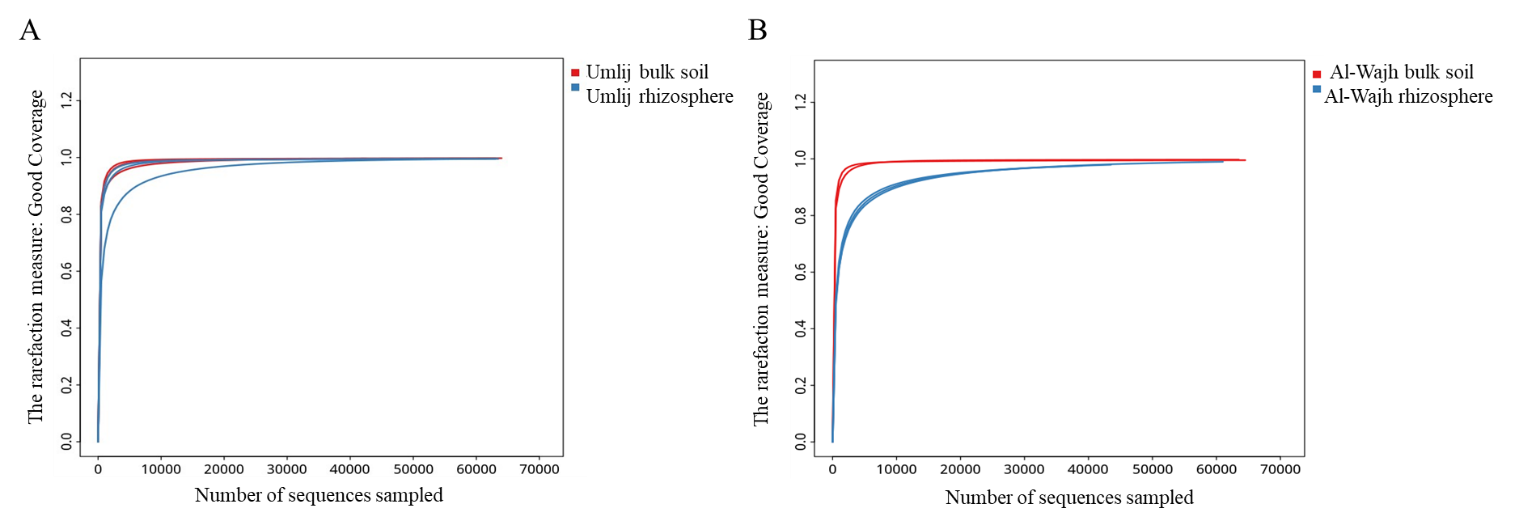


**Supplementary information Figure S2:**

The rarefaction curves computed for alpha diversity index (good coverage) of the observed OTUs of individual samples of the bulk and rhizosphere soils from Umluj and Al-wajh.
